# Cellular prostatic acid phosphatase (cPAcP) serves as a useful biomarker of histone deacetylase (HDAC) inhibitors in prostate cancer cell growth suppression

**DOI:** 10.1186/s13578-015-0033-y

**Published:** 2015-07-17

**Authors:** Yu-Wei Chou, Fen-Fen Lin, Sakthivel Muniyan, Frank C Lin, Ching-Shih Chen, Jue Wang, Chao-Cheng Huang, Ming-Fong Lin

**Affiliations:** Tissue Bank and BioBank, Kaohsiung Chang Gung Memorial Hospital, No. 123, Da-Pi Road, Niao-Song District, Kaohsiung, 833 Taiwan, ROC; Department of Biochemistry and Molecular Biology, College of Medicine, 985870 Nebraska Medical Center, University of Nebraska Medical Center, Omaha, NE 68198-5870 USA; Division of Urology, Department of Surgery, University of Arizona Medical Center, Tucson, AZ USA; Division of Medicinal Chemistry and Pharmacology, College of Pharmacy, The Ohio State University, Columbus, OH USA; Division of Oncology/Hematology, Department of Internal Medicine, University of Nebraska Medical Center, Omaha, NE USA; University of Arizona Cancer Center, St. Joseph’s Hospital and Medical Center, Phoenix, AZ USA; Department of Pathology, Kaohsiung Chang Gung Memorial Hospital, and Chang Gung University College of Medicine, Kaohsiung, Taiwan, ROC; Eppley Institute for Research in Cancer and Allied Diseases, University of Nebraska Medical Center, Omaha, NE USA; Department of Surgery/Urology, University of Nebraska Medical Center, Omaha, NE USA; School of Pharmacy, Kaohsiung Medical University, Kaohsiung, Taiwan, ROC

**Keywords:** Prostate cancer, Histone deacetylase inhibitor, Cellular prostatic acid phosphatase, Biomarker

## Abstract

**Background:**

Prostate cancer (PCa) is the most commonly diagnosed solid tumor and the second leading cancer death in the United States, and also one of the major cancer-related deaths in Chinese. Androgen deprivation therapy (ADT) is the first line treatment for metastatic PCa. PCa ultimately relapses with subsequent ADT treatment failure and becomes castrate-resistant (CR). It is important to develop effective therapies with a surrogate marker towards CR PCa.

**Method:**

Histone deacetylase (HDAC) inhibitors were examined to determine their effects in androgen receptor (AR)/cellular prostatic acid phosphatase (cPAcP)-positive PCa cells, including LNCaP C-33, C-81, C4-2 and C4-2B and MDA PCa2b androgen-sensitive and androgen-independent cells, and AR/cPAcP-negative PCa cells, including PC-3 and DU 145 cells. Cell growth was determined by cell number counting. Western blot analyses were carried out to determine AR, cPAcP and PSA protein levels.

**Results:**

cPAcP protein level was increased by HDAC inhibitor treatment. Valproic acid, a HDAC inhibitor, suppressed the growth of AR/cPAcP-positive PCa cells by over 50% in steroid-reduced conditions, higher than on AR/cPAcP-negative PCa cells. Further, HDAC inhibitor pretreatments increased androgen responsiveness as demonstrated by PSA protein level quantitation.

**Conclusion:**

Our results clearly demonstrate that HDAC inhibitors can induce cPAcP protein level, increase androgen responsiveness, and exhibit higher inhibitory activities on AR/cPAcP-positive PCa cells than on AR/cPAcP-negative PCa cells. Upon HDAC inhibitor pretreatment, PSA level was greatly elevated by androgens. This data indicates the potential clinical importance of cPAcP serving as a useful biomarker in the identification of PCa patient sub-population suitable for HDAC inhibitor treatment.

## Background

Prostate cancer is the most commonly diagnosed solid tumor and the second leading cancer death in United States. While the incidence in Chinese population is lower than in Western countries, it is rising rapidly and becomes one of the major cancer-related deaths in this region [1, 2]. Androgen-deprivation therapy (ADT) is the first line treatment for the advanced metastatic PCa by decreasing the production of androgens or the functions of AR with anti-androgen agents [3–5]. Unfortunately, most PCa will eventually still progress to castration-resistant prostate cancer (CR PCa) which represents an aggressive and incurable phenotype after a certain time period of treatment [4, 6]. Although new FDA approved therapies demonstrating increased survival benefits for CR PCa treatment exist, there are still no treatment modalities to inhibit the development of CR PCa [3].

Human prostatic acid phosphatase (PAcP), a 100 kDa glycoprotein of two subunits, is a prostate epithelium-specific differentiation antigen: one stays intracellularly, the cellular form (cPAcP), and the other secrets into seminal fluid, the secretory form (sPAcP), both encoded by the same gene [7, 8]. In normal males, circulating sPAcP activity is negligible. Interestingly, the serum activity of sPAcP is significantly elevated in PCa patients, especially in PCa patients with bone metastasis, and also correlated with tumor progression. sPAcP was thus utilized as a surrogate maker for the diagnosis and prognosis of PCa before the availability of prostate-specific antigen (PSA) despite the decreased expression of mRNA levels [9, 10]. sPAcP can also serve as an independent predictor of tumor recurrence following radical prostatectomy [7, 11]. Additionally, several lines of evidence show that cPAcP may serve as an excellent measure to elucidate the molecular mechanism of the relationship between androgens and tyrosine phosphorylation signaling involved in prostate cancer progression [8, 12–15]. cPAcP has been shown to be a negative growth regulator of prostate epithelia through its neutral protein tyrosine phosphatase (PTP) activity by dephosporylating p-Tyr of ErbB-2, which results in regulating androgen sensitivity [5, 12, 15]. Interestingly, cPAcP is involved in the effect of HDAC inhibitors on PCa cell growth suppression via its up-regulation for ErbB-2 dephosphorylation, and knockdown PAcP expression by shRNA reduces the degree of growth suppression by HDAC inhibitor [16]. Collectedly, cPAcP protein serves as a prostate epithelial differentiation marker and functions as a unique prostate-specific tumor suppressor [8].

Histone acetylation is regulated by a dynamic balance between histone acetyl transferases (HATs) and histone deactylases (HDACs), and involved in affecting the chromatin folding during gene expression [17, 18]. HDACs modulate transcriptional activity of hormonal receptors including AR, for example, by altering the stability of the transcriptional pre-initiation complex and/or modifying the chromatin structure. Many lines of evidence demonstrate HDACs over-expression or close association in multiple cancers, including PCa [3, 19]. HDAC inhibitors are epigenetic therapy agents targeting class I and/or class II histone deacetylases which alter not only histone and also non-histone protein function [6, 18]. HDAC inhibitors have been shown to mediate tumor cell differentiation, exhibit a dramatic inhibitory effect on tumor cell proliferation and death [3, 20]. HDAC inhibitors have therefore been considered to be a novel class of cancer treatment agents and a number of inhibitors have been entered into clinical trials for PCa therapy.

A biomarker such as a measurable molecular, cellular, or genetic parameter should indicate the biological or pathological conditions or pharmacological response to the treatments. Drug discovery and development in cancer research is rapidly approaching personalized or mechanism-based targeting therapy. The application of a suitable biomarker in the novel treatment could bring various advantages, such as the increasing potency, specific tumor selectivity, reducing toxicities and side effect profiles, improving the quality of patient’s life. HDAC inhibitors may serve as a novel class of anti-cancer agents; to develop a selecting biomarker for patient population who are suitable to HDAC inhibitor treatment requires further identification. In our previous study, we investigated the molecular target by HDAC inhibitors for exploring their potential of CR PCa therapy. We found that cPAcP expression is involved in growth suppression by HDAC inhibitors in AR-positive PCa cells, and HDAC inhibitor pre-treatment could increase androgen responsiveness of those PCa cells [16].

In this study, we tested the hypothesis that cPAcP can serve as a useful biomarker for identifying patients sensitive to HDACi treatments. We analyzed six different HDAC inhibitors which have shown inhibitory activities on tumor cell proliferation and/or viability or entered in various clinical trials, including valproic acid (VPA), sodium butyrate (NaB), suberoylanilide hydroxamic acid (SAHA), PxD 101, MS-275 and AR-42 [6, 16, 20–29] on six AR/cPAcP-positive and four AR/cPAcP-negative PCa cell lines. Our data provide strong evidence for the role of cPAcP expression in various HDAC inhibitors treatment in PCa cells. HDAC inhibitor treatment elevated cPAcP expression level and increased the androgen responsiveness of AR/cPAcP-positive PCa cells as shown by elevated PSA protein levels. Importantly, in steroid-reduced conditions mimicking clinical androgen deprivation therapy, AR/cPAcP-positive PCa cells were more sensitive to inhibitory efficiency of HDAC inhibitors treatment than AR/cPAcP-negative PCa cells. These results have important clinical impacts on identifying a useful biomarker for HDAC inhibitors toward advanced CR PCa treatment and also on predicting clinical treatment outcome.

## Results and discussion

Cellular prostatic acid phosphatase (cPAcP) is a unique prostate-specific tumor suppressor and its loss of expression is associated with prostate carcinogenesis [8]. The data also revealed that cPAcP is involved in regulating androgen-stimulated PCa cell growth, and its expression is associated with androgen-sensitive cell proliferation [7, 16]. Previously, we identified that cPAcP is one of molecular targets by HDAC inhibitors in PCa growth suppression. In HDAC inhibitor-treated AR-positive PCa cells, cPAcP is elevated and cell growth is suppressed; conversely, knockdown cPAcP expression by shRNA reduces the degree of growth suppression by HDAC inhibitors [16]. Furthermore, cPAcP in addition to AR plays a critical role in regulating androgen sensitivity of PCa cell proliferation. While AR is essential to androgen sensitivity; the presence of AR alone is not sufficient for androgen responsiveness of growth stimulation. For example, CR PCa cells still express functional AR but are androgen unresponsive. Instead, the expression of cPAcP in those cells is associated with androgen sensitivity. Since cPAcP expression is responsive to but not regulated by androgens, cPAcP level is not consistent with AR level. Importantly, upon VPA pretreatment, the androgen responsiveness of cells was increased, higher than control cells. As a clinical correlation, we selected cPAcP as a surrogate marker and further explored the role of cPAcP expression in PCa cell growth suppression by various HDAC inhibitors and examined whether HDAC inhibitors treatment will indeed alter androgen responsiveness of different PCa cells.

**Figure 1:**
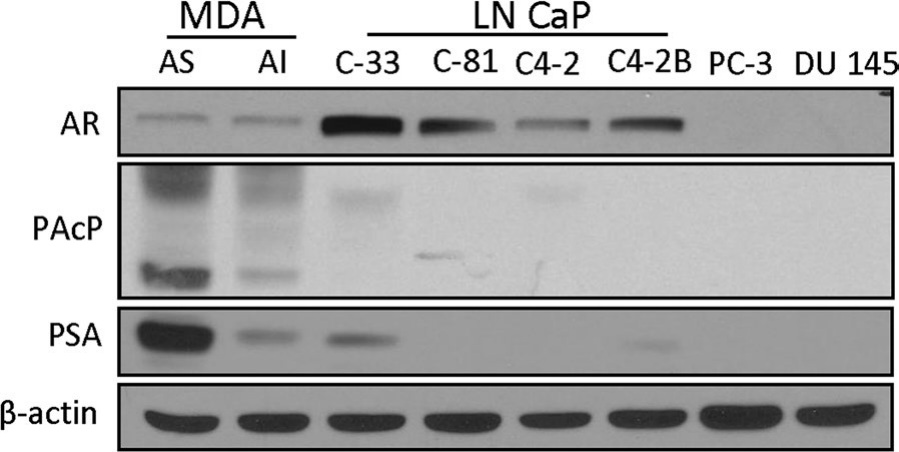
The basal expression levels of AR, cPAcP and PSA were determined in different PCa cell lines. LNCaP C-33/C-81, LNCaP C4-2/C4-2B, MDA PCa2B AS/AI, PC-3 and DU 145 cells that were plated in regular medium for 3 days and then changed with fresh medium for 1 day. The cells were harvested and the total protein was subjected to western blot analyses of functional proteins expression. β-Actin was analyzed and used as a loading control.

First, we analyzed the basal level of AR, PAcP and PSA proteins in different PCa cell lines. As shown in Figure 1, in regular culture condition, MDA PCa2b AS and AI cells, and LNCaP C-33, C-81, C4-2 and C4-2B cells all express AR protein. Among them, LNCaP cells had an overall higher levels of AR protein than MDA PCa2b cells, and LNCaP C-33 cells expressed the highest AR protein level among these cells examined (Figure 1), correlating with the degree of androgen-stimulated cell growth (data not shown). On the contrary, MDA PCa2b cells express higher levels of cPAcP than LNCaP cells (Figure 1) and have slower cell proliferation [30]. Furthermore, in MDA PCa2b and LNCaP cell models, cPAcP level decreased in AI cells, lower than that in the corresponding AS cells, respectively. The low PAcP level in AI LNCaP C-81, C4-2 and C4-2B could be seen upon prolonged exposure (data not shown). The PSA protein level is also decreased in AI cells. In comparison, PC-3 and DU 145 cells do not express a detectable level of AR, PAcP and PSA proteins (Figure 1) despite prolonged exposure (data not shown).

**Figure 2:**
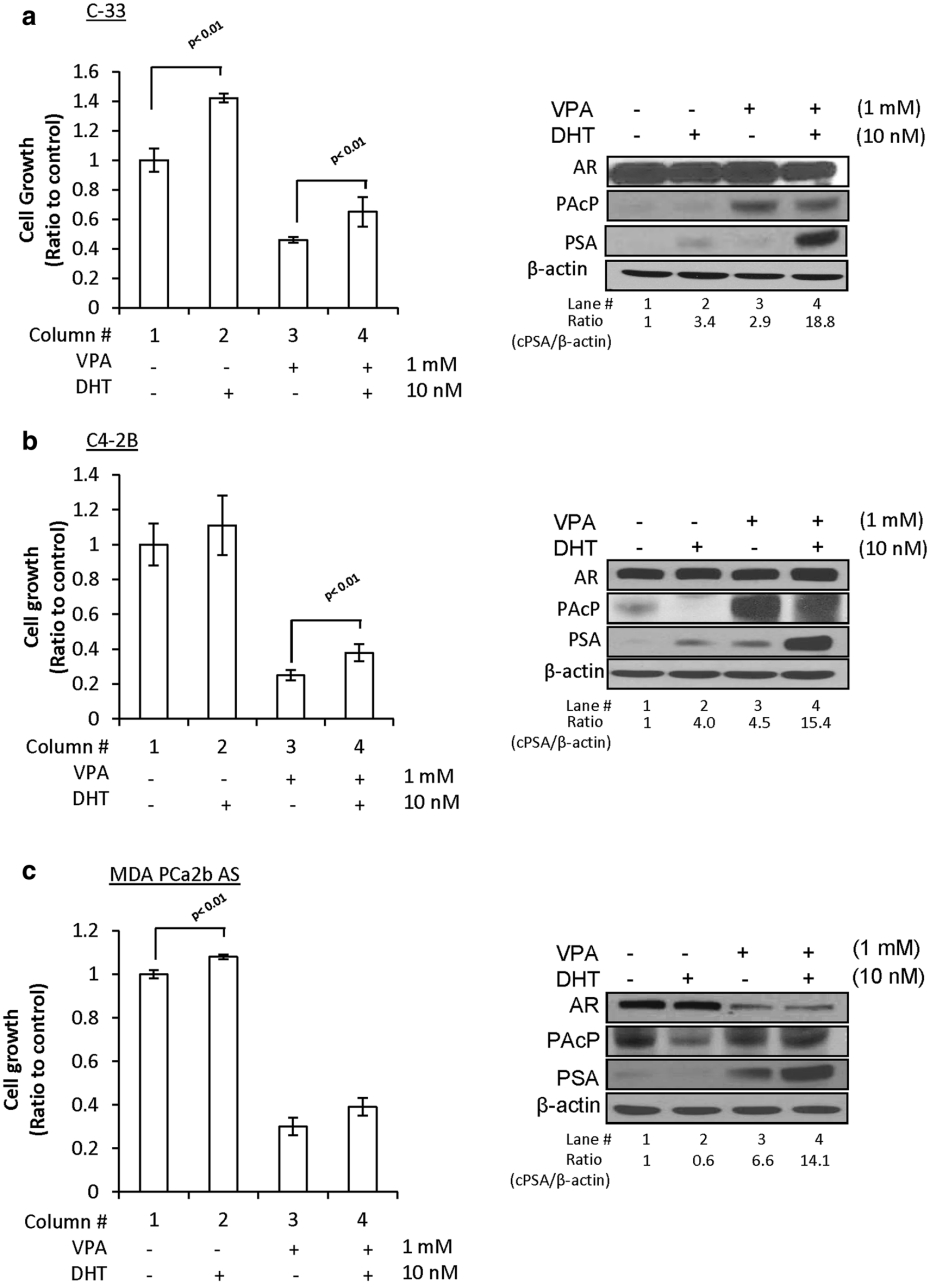
Effect of VPA on androgen responsiveness of PCa cell lines. **a** LNCaP C-33; **b** LNCaP C4-2B; **c** MDA PCa2B AS cells were seeded in 6-wells plate and then treated with 1 mM VPA or solvent for 48 h. Cell were then maintained in a steroid-reduced medium with or without 10 nM DHT for 2 days. Total cell number was counted. The ratio of cell growth was calculated by normalizing the cell number to that of control cells (*column* #*1*, *left panel*, n = 3×2). Total cell lysate proteins from 3-day DHT treatment were analyzed for cPAcP, PSA, AR protein. β-Actin was analyzed and used as a loading control (*right panel*).

We determined the effect of HDAC inhibitor on cPAcP protein level in AR-positive PCa cells and examined their relationship since cPAcP protein functions as a tumor suppressor in PCa cells. We first examined VPA effect on cPAcP protein level. As shown in Figure 2a–c, upon VPA treatment, cPAcP protein levels were greatly elevated in LNCaP C-33 and C4-2B cells, compared with control cells received the solvent alone (Figure 2a, b, Lane #3 vs. #1, right panel), which were decreased by subsequent DHT treatment (Figure 2a, b, Lane #4 vs. #3, right panel). cPAcP protein level had only a slight elevation in VPA-treated MDA PCa2b-AS cells (Figure 2c, Lane #3 vs. #1, right panel), which could be in part due to a very high basal level of cPAcP protein in those cells (Figure 1). Similar phenomenon was observed in LNCaP C-81, C4-2 and MDA PCa2B AI PCa cells (data not shown; [16]). Therefore, the observations on cPAcP protein levels that are increased in all VPA-pretreated PCa cells and then decreased by DHT treatment are inversely correlated with cell growth (Figure 2, Column #1 vs. #3, #3 vs. #4, left panel).

Since androgen sensitivity is an important clinical phenomenon; we examined VPA effect on the androgen responsiveness. Importantly, the PSA level was greatly elevated by 10 nM DHT in VPA-pretreated LNCaP cell lines by over 18-fold, higher than that of control cells without VPA-pretreatment with about fourfold increase (Figure 2a, b, Lane #4 vs. #2, right panel). In VPA-pretreated MDA PCa2b AS cells, PSA protein level was greatly elevated, which is further increased upon DHT treatment (Figure 2c, Lane #4 vs. #3, right panel); despite the fact that DHT alone only had a marginal effect on PSA protein level in the absence of VPA pretreatment, the similar trends were observed in LNCaP C-81, C4-2 and MDA PCa2B AI PCa cell lines (data not shown; [16]). In summary, our data show that VPA pretreatment can increase the degree of androgen sensitivity in cell proliferation by cell number counting and PSA protein level, an androgen-regulated marker, despite that in MDA PCa2b AS cells, VPA pretreatment only greatly enhanced DHT-increased PSA level (Figure 2c, Lane #3 vs. #4, right panel) but not cell growth (Figure 2c, Column #3 vs. #4, left panel). Furthermore, cPAcP protein level was indeed elevated by VPA treatment and then diminished by subsequent DHT treatment (Figure 2, Lane #3 vs. #4, right panels). Since VPA pretreatment greatly enhances DHT-upregulated PSA level, the data indicate that VPA can increase androgen responsiveness of AR-positive PCa cells.

**Figure 3:**
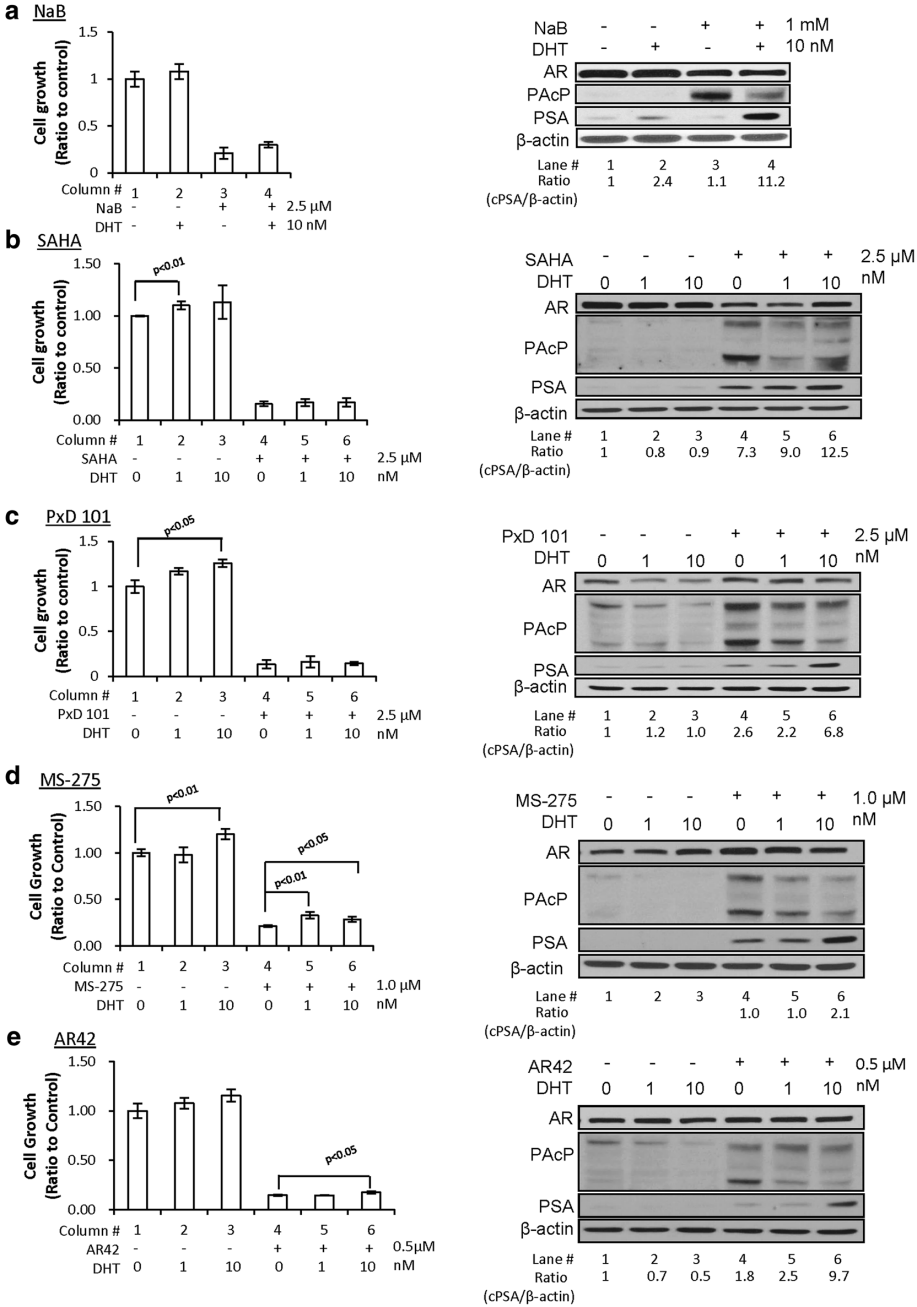
Effects of different HDAC inhibitors on androgen responsiveness of PCa cells. LNCaP C-81 cells were plated in 6-wells plate and then treated with **a** 1 mM NaB; **b** 2.5 µM SAHA; **c** 2.5 µM PxD101; **d** 1.0 µM MS-275; **e** 0.5 µM AR42 or solvent alone for 48 h. Cell were then maintained in a steroid-reduced medium with or without 1 and 10 nM DHT for 3 days. Total cell number was counted. The ratio of cell growth was calculated by normalizing the cell number to that of control cells (*column* #*1*, *left panel*, n = 3×2). Total cell lysate proteins were analyzed for cPAcP, PSA and AR protein. β-Actin was analyzed and used as a loading control (*right panel*).

We examined whether other HDAC inhibitors could similarly enhance the androgen responsiveness of PCa cells. LNCaP C-81 cells were used as the model for HDAC inhibitors pretreatment since C-81 cells exhibit many biochemical properties of CR PCa cells (16, 31, 32). C-81 cells were treated with HDAC inhibitors including NaB, SAHA, PxD101, MS-275 and AR42. Results of western blot analyses showed that cPAcP protein including the 38 kDa intermediate form and 50 kDa mature form were greatly elevated by all HDAC inhibitor treatments (Figure 3a–e, Lane #3 vs. #1, right panel) and were diminished by subsequently 1 and 10 nM DHT treatments, following the dose-dependent manner (Figure 3a, Lane #4 vs. #3, right panel; Figure 3b–e, Lane #5, 6 vs. Lane #4, right panel). Further analyses on Figures 2 and 3 reveal that the efficacy of HDAC inhibitor’s growth suppression is at least in part correlated with the degree of induced expression of cPAcP in addition to cPAcP basal level.

Furthermore, we validated DHT responsiveness by analyzing PSA protein level followed by semi-quantification in HDAC inhibitors-pretreated cells. In absence of DHT, PSA basal levels were greatly elevated by SAHA, PxD 101 and MS-275 treatments, and slightly increased in AR42-treated cells, but not significantly elevated in NaB-treated cells, respectively (Figure 3b–e, Lane #4 vs. #1; Figure 3a, Lane #3 vs. #1, right panel). Importantly, PSA expression levels were greatly elevated by 10 nM DHT in all HDAC inhibitor-pretreated cells by up to 12-fold of that in control cells without HDAC inhibitor pretreatment (Figure 3a, Lane #4 vs. #2; Figure 3b–e, Lane #6 vs. #3 right panel). However, the growth stimulation by DHT was only marginally increased in those HDAC inhibitors-pretreated cells comparing with control cells and only significantly increased in MS-275 and AR42 pretreated cells (Figure 3a, Column #4 vs. #3; Figure 3b–e, Column #5, 6 vs. #4, left panel). The data collectively indicate that HDAC inhibitors exhibit the significant efficacy of growth suppression and can enhance the androgen responsiveness of PSA levels. Nevertheless, the effect of HDAC inhibitors pretreatment on DHT-stimulated cell growth requires further investigation.

**Figure 4:**
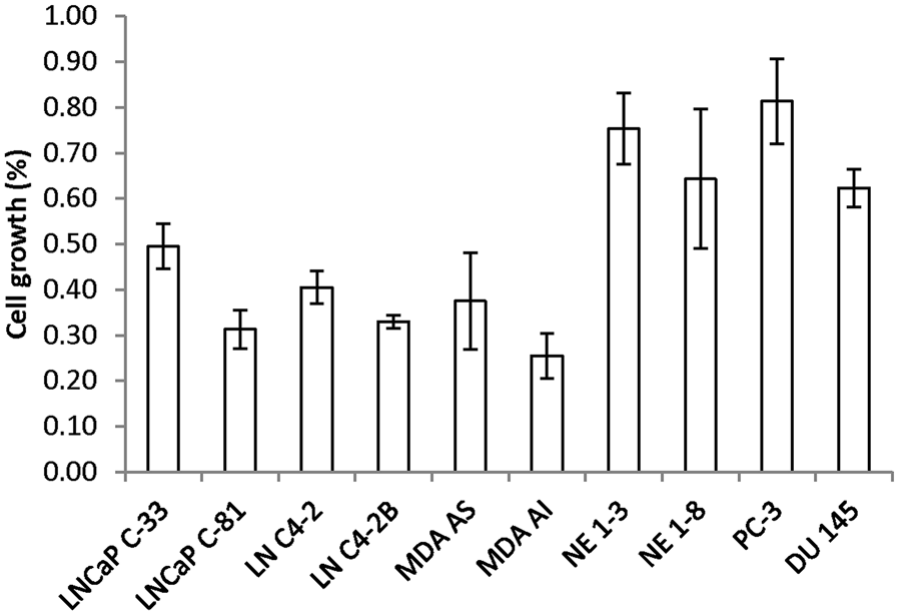
The growth inhibition of VPA treatment on various PCa cells. LNCaP C-33/C-81, LNCaP C4-2/C4-2B, MDA PCa2B AS/AI, NE 1-3/1-8, PC-3 and DU 145 PCa cell lines were plated in 6-wells plates and then treated with 1 mM VPA or solvent for 48 h. Cells were then maintained in a steroid-reduced medium for 3 days. Total cell number was counted. The ratio of cell growth was calculated by normalizing the cell number to that of control cells (n = 3×2).

Since the expression of cPAcP correlates with growth suppression by HDAC inhibitors, we investigated whether cPAcP protein level can serve as a useful biomarker of identifying the PCa patient sub-population who is potentially responsive to HDAC inhibitors treatment. To mimic the clinical situation of chemotherapy under androgen ablation conditions, cell growth suppression was determined in steroid-reduced (SR) conditions. Six AR/cPAcP-positive PCa cell lines and four AR/cPAcP-negative PCa cell lines including NE 1–3 and NE 1–8 cells, two neuroendocrine prostate cancer cell lines, were examined. After 2 days of VPA treatment, all PCa cells were maintained in SR medium for 72 h. Interestingly, the growth of those AR/cPAcP-positive PCa cells were in general significantly decreased by VPA treatment with more than 50% suppression; while the growth of AR/cPAcP-negative PCa cells was suppressed by less than 50% (Figure 4). The data indicate that cPAcP can potentially serve as a biomarker for HDAC inhibitor treatment with clinical benefits. Those patients can be more sensitive to HDAC inhibitor treatment, i.e., higher growth suppression and enhanced androgen responsiveness.

## Conclusion

In summary, the results of our study clearly show that in all HDAC inhibitors-treated AR/cPAcP-positive PCa cells, growth suppression by HDAC inhibitors is associated with elevated cPAcP protein level, and the androgen responsiveness of those PCa cells is also enhanced. PCa cells which exhibit endogenous cPAcP expression are more sensitive to HDAC inhibitors as shown by higher inhibitory efficiency with more than 50% compared with that of PCa without cPAcP expression by HDAC inhibitor treatments (Figure 4). Our data reveal that cPAcP can serve as a biomarker for identifying PCa patients who are sensitive to be treated by HDAC inhibitors. cPAcP can thus serve as a surrogate marker in PCa therapy, predicting clinical outcome to decrease the medical resources waste and improve the treatment efficacy. Furthermore, HDAC inhibitor-treated patients may re-gain their androgen responsiveness and thus are suitable to continue ADT treatment. HDAC inhibitors significantly increase PSA protein level while inhibit PCa cell proliferation, serum PSA cannot serve as a suitable marker for predicating the efficacy of treatments by HDAC inhibitors.

## Methods

### Materials

RPMI 1640 medium, gentamicin and trypsin/EDTA reagents were purchased from Invitrogen Corporation (Carlsbad, CA, USA). Fetal bovine serum (FBS), charcoal/dextran-treated, certified FBS was from Atlanta Biologicals (Lawrenceville, GA, USA). Acrylamide, protein molecular weight standard markers and Protein Estimation Kit were obtained from Bio-Rad (Hercules, CA, USA). The ECL reagent kit was purchased from Pierce Biotechnology Inc. (Rockford, IL, USA). Histone deacetylase inhibitors including sodium butyrate (NaB), valproic acid (VPA), 5α-dihydrotestosterone (DHT) and anti-β-Actin Ab (AC-15) were from Sigma (St Louis, MO, USA). Other HDAC inhibitors, including suberoylanilide hydroxamic acid (SAHA), AR42 and MS-275, were kindly provided by Dr. Ching-Shih Chen at the Ohio State University Comprehensive Cancer Center (Columbus, OH, USA). PxD101 was from Dr. Jue Wang at Medical Oncology section, University of Nebraska Medical Center (Omaha, NE, USA). Rabbit anti-human PAcP Ab (ATM-3) has been described previously [14, 15]. The respective Abs against androgen receptor (AR) and PSA were from Santa Cruz Biotechnology (Santa Cruz, CA, USA).

### Cell culture

Human prostate carcinoma cell lines including LNCaP, MDA PCa2b, PC-3 and DU 145 cells were originally purchased from the American Type Culture Collection (Rockville, MD, USA). LNCaP C4-2 and C4-2B cells were purchased from DIANON Company (Oklahoma City, OK, USA). LNCaP C-33/C-81, PC-3 and DU 145 cells were routinely maintained in the regular medium, i.e., phenol red-positive RPMI 1640 medium supplemented with 5% FBS, 2 mM glutamine and 50 µg/ml gentamicin. MDA PCa2b cells were cultured in BRFF-HPC1 medium containing 20% FBS, 2 mM glutamine and 50 µg/ml gentamicin. LNCaP C4-2 and C4-2B cells were grown in DMEM/F12 medium with 10% FBS, 2 mM glutamine, 50 µg/ml gentamicin, 1 mM sodium pyruvate, 2× vitamin C and 1× MEM non-essential amino acid. Cells were split once a week by trypsinization, which was defined as one passage.

The LNCaP PCa cell progression model was originally described by Lin et al. [31] and further characterized by Igawa et al. [32] with passage number less than 35 defined as C-33, passage numbers between 80 and 120 as C-81 cells. LNCaP C-81 cells exhibit many biochemical properties similar to the phenotype of advanced CR PCa, including the intracrine growth regulation, PSA secretion and rapid cell proliferation under steroid-deprived conditions. LNCaP C4-2/C4-2B cells also exhibit androgen independency of growth [33]. Similarly, MDA PCa2b-AI cells, the high passage MDA PCa2b cells, exhibit androgen-independent proliferation as described [12, 34, 35]. In this set of experiments, the passage numbers of MDA PCa2b-AI cells were between about 110 and 125.

### Effect of HDAC inhibitor pretreatment on the androgen responsiveness and cell growth suppression of PCa cell lines

Various PCa cell lines, including LNCaP C-33/C-81, LNCaP C4-2/C4-2B and MDA PCa2b AS/AI cells were plated with 3 × 10^4^, 3 × 10^4^ and 1 × 10^5^ cells/well, respectively, in 6-well plates in regular medium for 3 days and then treated with 1 mM VPA or different concentrations of HDAC inhibitors as specified in each experiment for 2 days. Control cells were treated with solvent alone. Subsequently, cells were maintained in a steroid-reduced medium minus or plus 1 and 10 nM DHT for 2 days. Cells were harvested and cell numbers were counted using a Cellometer Auto T4 Image-based cell counter (Nexcelom Bioscience). All experiments were performed in triplicate and repeated at least twice. Results shown were an average or a representative from two or three sets of independent experiments. Cells were lysed for analyzing cPAcP, AR and PSA protein expression. β-Actin was used as a loading control.

To determine the effect of cPAcP on PCa cell growth suppression by HDAC inhibitors, LNCaP (C-33/C-81, C4-2/C4-2B), MDA PCa2B AS/AI, NE1-3/1-8, PC-3 and DU145 PCa cells were plated in 6-well plates in regular medium for 3 days, treated with 1 mM VPA for 2 days and then maintained in steroid-reduced medium for 3 days. Control cells were treated with solvent alone. The cell numbers were counted. The ratio of cell growth was calculated by normalizing the number of experimental cells to that of control cells.

### Immunoblotting

For analyzing cellular protein levels, subconfluent cells were harvested by scraping. The cell pellet was rinsed with ice-cold 20 mM HEPES-buffered saline (pH 7.0) and then lysed in ice-cold cell lysis buffer (20 mM Tris–HCl, pH 7.5, 150 mM NaCl, 1 mM EDTA, 1% Nonidet P-40) containing protease and phosphatase inhibitors and the total lysate protein was prepared upon centrifugation. An aliquot of total cellular lysate having 50–120 µg protein was subjected to electrophoresis on SDS–polyacrylamide gels (7.5–12% acrylamide) and then transferred to nitrocellulose membrane for western blot analyses. The membrane filter was blocked by 5% skim milk and subsequently incubated with appropriate primary and secondary Ab. The proteins of interest were visualized by an ECL detection system. For re-probing, the membranes were stripped with a stripping buffer for 30 min at 50°C, blocked and re-hybridized with specific Abs [16].

### Statistical analysis

Each experiment was performed in duplicate or triplicates and repeated at least twice as independent experiments, as specified in each figure legend or experiment design, and the mean and standard error values were calculated. The significance of difference (*p* value) was calculated using independent Student t test and the *p* value less than 0.05 was considered as significant.

## Abbreviations

PCa: prostate cancer
PAcP: prostatic acid phosphatase
HDAC: histone deacetylase
